# Brain Mapping the Effects of Chronic Aerobic Exercise in the Rat Brain Using FDG PET

**DOI:** 10.3390/jpm12060860

**Published:** 2022-05-25

**Authors:** Colin Hanna, John Hamilton, Eliz Arnavut, Kenneth Blum, Panayotis K. Thanos

**Affiliations:** 1 Behavioral Neuropharmacology and Neuroimaging Laboratory on Addictions, Department of Pharmacology and Toxicology, Clinical Research Institute on Addictions, Jacobs School of Medicine and Biosciences, University at Buffalo, Buffalo, NY 14203, USA; cshanna2@buffalo.edu (C.H.); jh268@buffalo.edu (J.H.); elizarna@buffalo.edu (E.A.); 2 Graduate College, Western University Health Sciences, Pomona, CA 91766, USA; drd2gene@gmail.com; 3 Department of Psychology, State University of New York at Buffalo, Buffalo, NY 14203, USA

**Keywords:** rat, 18F-FDG Fluorodeoxyglucose, positron emission tomography, aerobic exercise, glucose metabolism, Statistical Parametric Mapping

## Abstract

Exercise is a key component to health and wellness and is thought to play an important role in brain activity. Changes in brain activity after exercise have been observed through various neuroimaging techniques, such as functional magnetic resonance imaging (fMRI) and positron emission tomography (PET). The precise impact of exercise on brain glucose metabolism (BGluM) is still unclear; however, results from PET studies seem to indicate an increase in regional metabolism in areas related to cognition and memory, direction, drive, motor functions, perception, and somatosensory areas in humans. Using PET and the glucose analog [18F]-Fluorodeoxyglucose (18F-FDG), we assessed the changes in BGluM between sedentary and chronic exercise in rats. Chronic treadmill exercise treatment demonstrated a significant increase in BGluM activity in the following brain regions: the caudate putamen (striatum), external capsule, internal capsule, deep cerebellar white matter, primary auditory cortex, forceps major of the corpus callosum, postsubiculum, subiculum transition area, and the central nucleus of the inferior colliculus. These brain regions are functionally associated with auditory processing, memory, motor function, and motivated behavior. Therefore, chronic daily treadmill running in rats stimulates BGluM in distinct brain regions. This identified functional circuit provides a map of brain regions for future molecular assessment which will help us understand the biomarkers involved in specific brain regions following exercise training, as this is critical in exploring the therapeutic potential of exercise in the treatment of neurodegenerative disease, traumatic brain injury, and addiction.

## 1. Introduction

Exercise is a well-established promotor of health and well-being. It has been found to improve physiological, psychological, and neurological functions across varying demographic variables. Additionally, sedentary behavior has been shown to increase the risk for adverse health effects, including diabetes, cardiovascular disease, and cancer [1]. As sedentary activity increases in classroom and workplace settings, researchers have begun to consider physical inactivity as a serious public health issue [2]. An extensive amount of research has shown that exercise interventions can be used for preventative health measures, as well as treatment/alleviation of adverse symptoms. It is well-known that exercise can have important benefits in treating many diseases, including cardiovascular disease [3,4,5], depression [6], anxiety [7,8], and traumatic brain injury [9,10,11,12]. Similarly, exercise has been shown to have therapeutic potential in neurological disease, and it has been shown that exercise can increase cortical thickness in brain regions associated with spatial orientation, memory, and vestibular processes [13]. In neurodegenerative disease, symptom attenuation following exercise interventions has been shown in Alzheimer’s disease [14,15]. Lastly, previous research has shown aerobic exercise to be an effective supplement to medications for patients with attention deficit hyperactivity disorder (ADHD), with post-exercise changes observable in both the frontal and temporal cortical regions [16,17,18,19]. In addition, aerobic exercise has shown great promise, as both the prevention and treatment of drug abuse [20,21,22,23,24,25,26,27,28].

To gauge how exercise influences specific brain regional activity, PET and 18F-Flurodeoxyglucose ([18F]-FDG) was used to assess brain glucose metabolism (BGluM) in the rat brain. FDG PET is commonly used in both humans [29,30] and rodents [31,32,33] to evaluate functional brain connectivity. Previous clinical research has shown that aerobic exercise increased BGluM in the left posterior entorhinal cortex, the left superior temporal gyrus, the right superior temporopolar area the occipital cortex, the premotor cortex, and the cerebellum in adults [34,35].

Further investigation is needed in the subject of BGluM and exercise. An understanding of the baseline effects of exercise could help determine how and why different types of exercise could be used to treat specific disorders. The present study examined the effects of chronic treadmill exercise on BGluM in rats compared to sedentary controls, using18F-FDG PET to determine the specific brain regions impacted.

## 2. Experimental Procedure

### 2.1. Animals

Young-adult female rats (n = 13; mean weight = 187.4 g) at eight weeks of age obtained from Taconic (Taconic, Hudson, NY) were individually housed in open-top cages at temperatures of 22.0 °C ± 2.0 °C with a 12 h reverse light/dark cycle (dark: 8:00 am to 8:00 pm). Rats were given unlimited access to food and water and were handled daily. This experiment was conducted in accordance with the National Academy of Sciences Guide for the Care and Use of Laboratory Animals (1996) and approved by the University at Buffalo Institutional Animal Care and Use Committee.

### 2.2. Exercise Regimen

**Figure 1:**
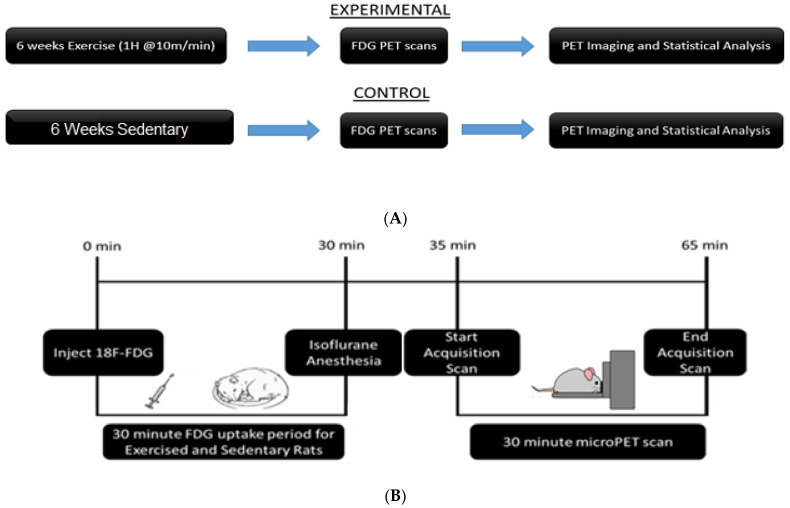
Experimental timelines: (**A**) Animals were divided into exercise and sedentary groups. Exercise animals received 6 weeks of exercise, while sedentary animals remained in their home cages. All animals underwent PET scans after the conclusion of 6 weeks. (**B**) Timeline of PET procedure: Animals were injected with [18F]-Fluorodeoxyglucose (FDG) via intraperitoneal injection. They were returned to their home cages for a 30-min uptake period. At the end of the uptake period, animals were anesthetized and placed in the bed of the PET R4 tomograph machine. PET scans lasted 30 min. After the scan, animals were recovered and returned to their home cages.

Rats were allowed one week to habituate to their environment. A customized treadmill, divided into six Plexiglas running lanes and enclosed by a piece of sheet metal, was used for exercise running, as previously described [21,23,27]. All exercise subjects (n = 6) received the same exercise regimen. Briefly, treadmill exercise was conducted during the rats’ dark cycle, between 10:00 am and 2:00 pm. Running began at 10 min/day at a steady rate of 10 m/min. The speed was held constant, and the exercise time was increased by 10 min/day until the peak rate of 60 min was reached. The animals were given a 10 min break after the first 30 min of running. Exercised rats maintained this exercise regimen, five days per week, for six weeks. The total distance ran after the six-week regimen was approximately 16.5 km [21]. Sedentary rats (n = 7) stayed in their home cages and received no treadmill exercise [21,26]. A complete experiment timeline can be seen in Figure 1.

### 2.3. Positron Emission Tomography (PET) Imaging

PET scans were conducted two weeks after the completion of the exercise regimen. In order to normalize baseline blood glucose levels, all rats experienced food restriction for 8 h before PET scans. Rats were given 500 ± 115 μCi of 18F-FDG via intraperitoneal injection. A 30-min uptake period followed the injection, in which the animals were allowed to freely roam around in their environment.

Rats were anesthetized immediately after the uptake period. Anesthesia was performed by using 3% isoflurane (maintained on 1% throughout the duration of the PET scan). Rats were secured on the bed of the scanner and scanned for 30 min, as per standard imaging protocol. Scans were recorded by using a PET R4 tomograph (Concorde CTI Siemens), which has a transaxial resolution of 2.0 mm full width at half maximum and a transaxial field view of 11.5 cm, as previously described [31]. After the rats were awakened, they were returned to their home cages and given food and water.

### 2.4. Image and Statistical Analysis

**Figure 2:**
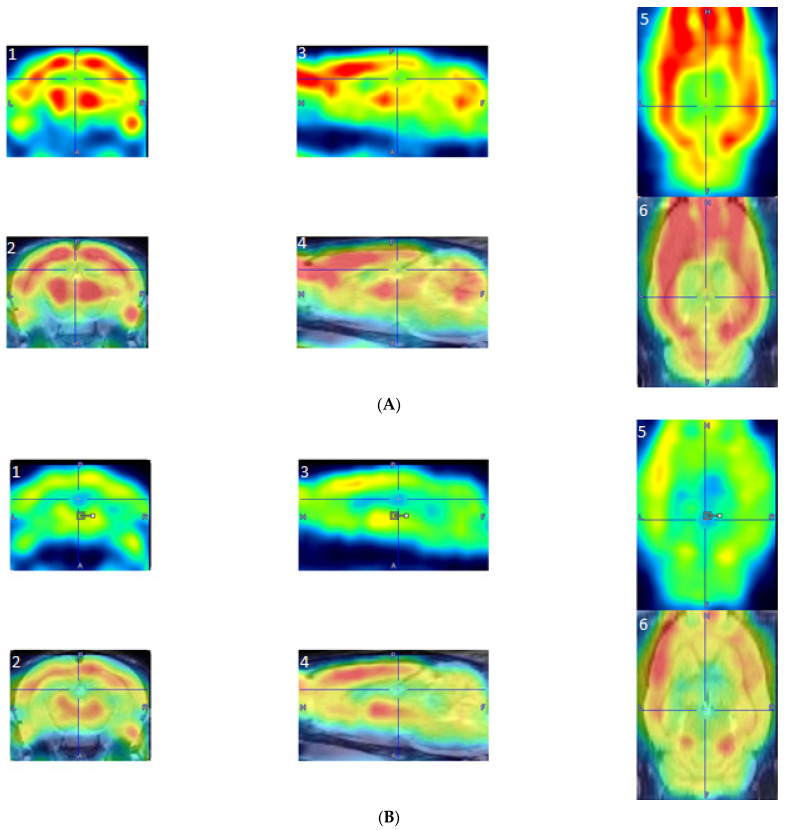
Examples of reconstructed PET images manually co-registered to an fMRI template. (**A**) PET image from an exercised rat: A1, Coronal PET image, exercised rat; A2, coronal co-registered image, exercised rat; A3, sagittal PET image, exercised rat; A4, sagittal co-registered image, exercised rat; A5, horizontal PET image, exercised rat; and A6, horizontal co-registered image, exercised rat. (**B**) PET images from a sedentary rat: B1, coronal PET image, sedentary rat; B2, coronal co-registered image, sedentary rat; B3, sagittal PET image, sedentary rat; B4, sagittal co-registered image, sedentary rat; B5, horizontal PET image, sedentary rat; and B6, horizontal co-registered image, sedentary rat.

PET scans were reconstructed via the MAP algorithm (15 iterations, 0.01 smoothing value, 256 × 256 × 256 resolution). Scans were manually co-registered onto a rat brain MRI template (63 slices) with Paxinos and Watson stereotaxic coordinates [36] in PMOD imaging software (version 2.85, PMOD Technologies). Scans with poor image quality were omitted from the analysis. Co-registered PET images can be seen in Figure 2. The aligned scans were then automatically co-registered and spatially normalized in MatLab Software (MatLab, R2018b). Statistical Parametric Mapping (SPM8) software was used to find significant differences in BGluM between exercised and sedentary rats. Proportional normalization with overall grand mean scaling was used in the SPM analysis. A two-sample t-test was conducted to analyze significant differences in clusters (significant voxel threshold, K > 50, *p* < 0.001). Cluster images were again fit to a rat brain MRI template, using AMIDE software (Stanford University), as previously described [31]. Clusters representing activation, or significantly greater BGluM in the exercise group, are indicated in the hot scale (red, yellow, and white clusters). Activated clusters were mapped and labeled by using “The Rat Brain in Steriotaxic Coordinates” atlas [36].

## 3. Results

**Figure 3:**
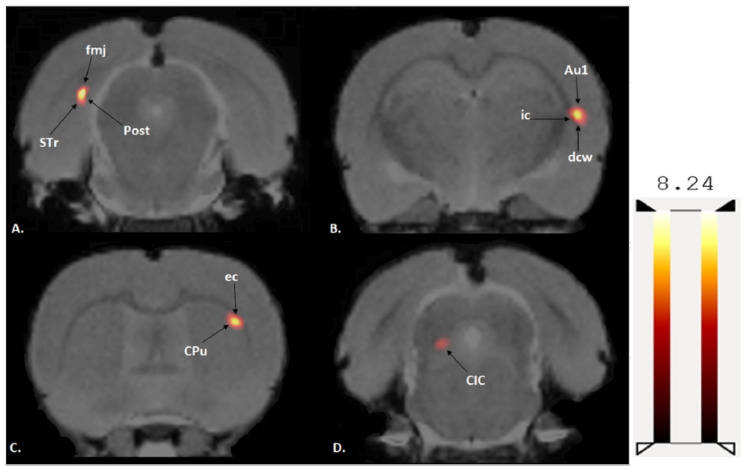
Coronal PET images showing brain regions with significant (*p* < 0.001, df = 11, and K > 50) differences in brain glucose metabolism (BGluM) between exercised and sedentary rats. Hot scale clusters illustrate BGluM activation. The value 8.24 represents peak activation level, as expressed by the t-value: (**A**) fmj, Post, Subiculum, and STr; (**B**) ic, dcw, and Au1; (**C**) CPu and ec; and (**D**) CIC.

A two-sample t-test revealed that exercised rats showed significantly increased BGluM (*p* < 0.001 df = 11), K > 50) compared to sedentary rats in the following regions: caudate putamen (striatum) (CPu), external capsule (ec), internal capsule (ic), deep cerebellar white matter (dcw), primary auditory cortex (Au1), forceps major of the corpus callosum (fmj), postsubiculum (Post), subiculum transition area (STr), and the central nucleus of the inferior colliculus (CIC).

(Exercise > Sedentary; Table 1 and Figure 3) BGluM activation is mapped in the brain (*p* < 0.001, df = 11). Activated regions are indicated in the hot scale. There was no significant inhibition in BGluM observed in exercise rats when compared to the sedentary rats (*p* > 0.05).

## 4. Discussion

These data help us determine how exactly specific brain areas are influenced by exercise. The effects of chronic aerobic exercise on BGluM have not been thoroughly investigated. While some human data exist, there are potential confounds and limitations posed by a lack of a controlled environment. The present preclinical study controlled for age, weight, environment (housing, care, and nutrition), and treatment (prescribed exercise or sedentary). Therefore, we can be certain that the regional changes in BGluM that are reported are exclusive to aerobic exercise. The activated brain regions include the Cpu, ec, ic, dcw, Au1, fmj, Post, STr, and CIC. Here, we describe the roles of the identified brain regions, how they react in concert with exercise, and how these regions are involved according to the literature in various diseases. This allows us to understand the present findings in the context of the literature on exercise neuroscience and disease pathology. We assert that these reported activated clusters are not influenced by stress of the exercise paradigm used, as past findings show that chronic exercise using this model did not result in increased levels of corticosterone in rats [23]. One limitation of this study was that rats placed daily in the treadmill may be an enriching environment (see limitations listed in conclusions paragraph).

**Figure 4:**
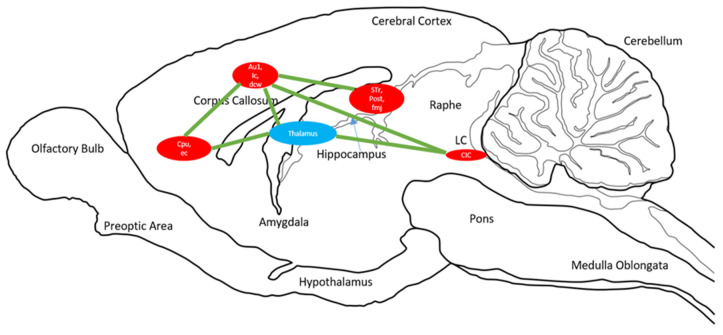
Sagittal drawing of overall brain circuits of brain glucose metabolism activation in response to chronic aerobic exercise. Significant clusters identified for *p* < 0.001, K > 50. Circle size is representative of cluster size. The blue circle is the thalamus, a relay point for many of the clusters shown in red. Green lines indicate activation circuits.

We also discuss a hypothesized circuitry of these clusters. The SPM analysis reveals clusters which are mapped on the brain. The circuit presented is simply the representation of all of these clusters throughout the brain that showed a similar BGluM activation in response to exercise as compared to sedentary scans. This approach has been previously used with FDG PET imaging [31,32] and further summarizes the results of this functional imaging (Figure 4).

### 4.1. CPu Activation

We found that aerobic exercise induced BGluM activation in the CPu (and the same cluster included part of the ec). The CPu is a major component of the basal ganglia and striatum. Its many functions include sensorimotor cognition, behaviors, habits, and premotor functions [37,38]. The striatum is primarily known to play a role in voluntary skeletal movements [39]. The CPu is also involved in planning movements, but it also plays an emotional role including motivation [39]. Dysfunction of the CPu is associated with several psychiatric disorders, such as obsessive compulsive disorder, ADHD, and bipolar disorder [39]. In addition, the CPu is involved in motor functions and cognition [40]. Dysfunction of the CPu is noted in many diseases, including Parkinson’s disease, Huntington’s disease, Alzheimer’s, depression, and autism [40].

Rodent electrophysiological recordings have shown neuronal discharge during movement in the basal ganglia [41]. Exercised rodents have also been shown to display an increase in dopaminergic activity in the CPu [42]. Changes in basal ganglia function are well documented in studies on Parkinson’s disease and other movement disorders [43]. The influence of exercise on Parkinson’s disease is supported by animal models. Four weeks of treadmill training was found to improve symptoms of Parkinson’s disease in rats [44]. This included changes in gait, walking speed, and limb/toe control. Temporary recovery in corticostriatal pathway synaptic plasticity was also found after exercise [44]. In humans, multiple studies have found that treadmill exercise increases striatal dopaminergic binding in patients with Parkinson’s disease [45,46].

Progressive resistance training is associated with an increase in functional connectivity between the caudate and the left inferior parietal lobe, the bilateral frontal lobes, and the right insula in patients with multiple sclerosis (MS) [5,47]. Increases in functional connectivity between the CPu and the left inferior parietal region were negatively correlated with symptoms of severe fatigue in MS patients [47]. In further support of the CPu response to exercise, caudate volume was found to correlate significantly with walking-task performances in MS patients [48]. This is supportive of the influence by the CPu in movement. Measurements of this and other basal ganglia areas may be signifiers of treatment progress in movement disorders, in which exercise holds a lot of therapeutic potential.

### 4.2. Auditory Processing Areas

Interestingly, we found a significant increase in BGluM in two areas associated with auditory processing: the CIC and Au1. The pathway between the CIC and the Au1 is well documented in animals and in humans [49,50,51,52,53]. Auditory processing pathways play a role in sensorimotor cognition as signals are relayed through the brainstem [54]. This follows the logic of the “transient hypofrontality hypothesis”, which poses a post-exercise redistribution of metabolic resources from frontal regions to sensorimotor regions [55,56].

The Au1 is a part member of the higher-order regions in the cerebral cortex [57]. It is a temporal region involved in the spatial localization of sound and integration of sonic information with other sensory systems [58]. In rats, the Au1 has been found to play a role in memory and stress responses via the thalalamo-cortico-amygdalar pathways [59]. Additionally, the Au1 has been reported to receive cholinergic input from the CPu in rats [60], and this coincides with our findings of dual activation of the Au1 and CPu. The Au1 cluster also included activation of the ic and dcw.

The CIC is a part of the midbrain, which relays auditory signals from the inner ear to the auditory cortex and the medial geniculate nucleus of the thalamus [61]. The activation of the CIC might indicate the inhibition of the thalamus via GABA neurons in rats [62]. Of the projections from the inferior colliculus to the thalamus (involved in somatosensory integration and motor control), 40% are GABAergic [62,63]. Known mostly for the relaying of sonic information, the inferior colliculus has also been speculated to be involved in the integration of multimodal sensory integration (including visual information), attention, and reflexes, thus supporting the findings on decreased reaction times reported above [62,64,65,66].

Less is known about the relationship between aerobic exercise and auditory processing on a neuroanatomical level, and there is little prior data that exercise activates the CIC and the Au1 in rodents. Studies have shown that exercise has an influence on reactions to auditory cues in humans and animals, with most evidence pointing to post-exercise improvements in auditory reactions. Aerobic exercise has been shown to improve reaction times and decrease errors in auditory discrimination tasks in perimenopausal women [66]. A 2013 study found that, among 50 healthy adult subjects, regular aerobic exercise significantly decreased reaction times in an audio-cued labeling task compared to controls who did not exercise regularly [65]. Additionally, in rats, six weeks of swimming exercise resulted in improved auditory memory, as indicated by a decrease in response time to auditory cues [64]. The evidence reported above supports the claim that exercise influences auditory processing, but further evidence is needed for a more complete neuroanatomical understanding.

Future research should investigate this complex further to see if exercised rats show the same patterns in areas of auditory processing. This proposition would be supported if the exercised rats are more sensitive to auditory stimulation or are more proficient in completing auditory tasks.

### 4.3. Activation of Sub-Hippocampal Regions

The STr and Post showed a significant increase in BGluM after 6 weeks of aerobic exercise. These areas are referred to as sub-hippocampal regions [67]. The subiculum is known to play a role in communication with subcortical areas and is involved in behavior [68]. Known to receive memory-related input and output, the circuitry and topography of the subiculum suggest its ability to differentiate stimuli from the hippocampus and relay the information to the thalamus and diencephalon [69]. The Post is known to play a role in head direction control and spatial coordination [70]. To support this, lesions of the Post have resulted in impairments of spatial memory and navigational tasks [71].

Activation of these hippocampal areas is consistent with the literature on exercise and hippocampal functioning. The hippocampus is known to play a role in learning and memory in both humans and animals [72,73,74]. Many studies confirm that the rodent hippocampus responds to exercise. Moderate treadmill exercise increased tyrosine phosphorylation of proteins related to plasticity in the rat hippocampus [75], along with brain-derived neurotropic factors immediately after acute moderate exercise [75]. In another study, vertical ladder resistance training was found to significantly increase 1/insulin growth factor receptor concentration in the hippocampus of adult rats compared to sedentary controls. In correlation with this finding, the exercised rats showed significant improvement in the passive avoidance memory task [76].

A novel study explored how proteins released in the bloodstream after exercise influence neurogeneration in the Dentate Gyrus of the hippocampus in mice [77]. Selenoptotein P, an antioxidant that is released into the blood after exercise, was shown to promote neurogenesis in both hippocampal stem cell cultures and in mice models. This protein triggers a cascade of activities, such as neurogenesis and proliferation, in the Dentate Gyrus of the hippocampus. Selenium increases via dietary supplementation and hippocampal infusion are coupled with greater spatial learning and improved memory [77].

The effects of exercise on the hippocampus are of great translational value to neuroscientists due to their potential to influence psychological and cognitive functioning. Aerobic exercise increased hippocampal volume and improved memory function in older adults, while the control group showed a decrease in hippocampal volume [78]. MRI studies show that the degeneration of hippocampal subfields (such as the subiculum) is associated with cognitive decline related to Alzheimer’s disease [79]. High-intensity resistance exercise can improve cognitive performance and offer protection from neurodegeneration and volume loss [79]. Our findings suggest that BGluM might play a role in the plasticity of the hippocampus, influencing features such as connectivity and changes in volume.

An 18F-FDG micro-PET study found BGluM inhibition in the inferior colliculus and the hippocampus in rats after a forced swim test [80]. This is contrary to our findings in both structures. However, in the case of the forced swim test, animals were only placed in a water tank once to measure the effects of acute stress on BGluM. Future studies might observe how repeated exposure to the forced swim test (training) might impact BGluM.

### 4.4. Increased White Matter Activity

Previous findings suggest that neuronal activity provokes subsequent myelination and oligodendrocyte activity [81,82]. Additionally, FDG PET studies have observed metabolic white matter changes in neurological diseases [83,84] and that glucose uptake in white matter is, while less than gray matter is, still significant [85].

White-matter structures in our clusters included the fmj, ic, ec, and dcw. The fmj is a white-matter structure whose fibers play a role in bridging the two occipital hemispheres through the corpus callosum [86]. Past research involving diffusor tensor imaging showed an increase in mean diffusivity in the left forceps major after 25 older adults were subjected to six months of aerobic exercise [87,88]. The present results showed an increase in activity in the ic and a white-matter structure on the inferomedial part of each cerebral hemisphere [89]. Its role includes afferent and efferent signaling to the cerebral cortex and separation of the caudate and thalamus medially from the putamen and globus pallidus laterally [90]. In deaf children, aerobic exercise was found to increase mean diffusivity in the left anterior limb of the ic [91]. The ec was shown to metabolically respond to the exercise treatment. The ec is a bundle of white matter fibers that surround the putamen. It is thought to receive input from prefrontal areas [92,93]. When damaged, the ec and other putamen-surrounding structures have been shown to slow and restrict walking speed in humans [94].

Evidence suggests that exercise has regenerative and rehabilitative effects by promoting myelination. One study showed that exercise had restorative effects in induced cerebral ischemia in rats [95]. Treadmill exercise significantly reduced the volume of infarction, decreased the size of damaged brain tissue, and increased repair-related myelination in the damaged areas. Exercised rats also showed an upregulation of myelin basic protein [95]. Glial and white matter responses are known to play a role in brain repair [96]. Exercise is also known to induce reparative effects in the brain. Six weeks of treadmill exercise has been shown to induce reparations in the prefrontal cortex (PFC), an area known to decrease in glial presence in both depressed patients and animals [97]. After exposure to chronic unpredictable stress, six weeks of treadmill running was shown to increase myelin basic protein, CNPase+ oligodendrocytes, and Olig2+ oligodendrocytes in the medial PFC of male rats [98]. This is suggestive of the reparative and modulatory mediators that are induced by aerobic exercise, coupled by increases in glial activation and myelination.

### 4.5. Conclusions

The results detail the effects of exercise training on BGluM. We report an increase in BGluM in brain areas associated with auditory processing (Au1 and the CIC), areas associated with memory (sub-hippocampal areas: Post and STr), and striatal activity. These results support the therapeutic potential of exercise in diseases related to the basal ganglia and can stimulate the brain circuitry to temporal areas from the CPu. Results also support that exercise training stimulates brain activity in brain areas involved in communication with cortical areas (increase in Au1 activity by the CPu). Lastly, exercise training increased activation in white matter areas, suggesting an increase in myelination. Some limitations of this study include the possibility that the reactivity of the reported brain regions to exercise may be species-specific. Lastly, daily treadmill exercise may be enriching to the rats, as it might contribute to changes in brain metabolism unaccounted for by exercise alone. These caveats give grounds for further research into BGluM, FDG PET, and exercise neuroscience. These data support the idea that exercise may be an important therapeutic adjunct to support learning and memory function and in initiating repair following neurological injury.

## Figures and Tables

**Table 1:** Brain regions where there was a significant brain glucose metabolism (BGluM) activation effect between exercised and sedentary rats at (*p* < 0.001, df = 11) voxel threshold K > 50. Coordinates in stereotaxic space (medial–lateral, anterior–posterior, and dorsal–ventral) are provided for cluster location. The t-values and z-scores were calculated from the average BGluM of all voxels within the significant clusters. The number of voxels in the significant clusters is noted “KE”, voxel size 0.2 mm isotropic. Different colors represent separate clusters.

BGluM in Overlapping Brain Region	Medial–Lateral (mm)	Anterior–Posterior (mm)	Dorsal–Ventral (mm)	t-Value	Z-Score	KE
CPu	4.0	−0.4	4.2	7.03	4.24	735
ec						
ic	5.4	−3.8	5.0	7.56	4.42	735
dcw						
Au1						
fmj	−4.0	−7.4	3.8	8.24	4.57	71
Post						
STr						
CIC	−1.8	−8.2	5.2	5.1	3.58	64

## Data Availability

Data is available from corresponding author if needed.
